# Satellite imagery for high-throughput phenotyping in breeding plots

**DOI:** 10.3389/fpls.2023.1114670

**Published:** 2023-05-16

**Authors:** Francisco Pinto, Mainassara Zaman-Allah, Matthew Reynolds, Urs Schulthess

**Affiliations:** ^1^ Global Wheat Program, International Maize and Wheat Improvement Center (CIMMYT), Texcoco, Mexico; ^2^ Global Maize Program, International Maize and Wheat Improvement Center (CIMMYT), Southern Africa Regional Office, Harare, Zimbabwe; ^3^ CIMMYT-China Wheat and Maize Joint Research Center, Agronomy College, Henan Agricultural University, Zhengzhou, China

**Keywords:** high-throughput phenotyping, satellite, wheat, maize, breeding, normalized difference vegetation index, optimized soil adjusted vegetation index

## Abstract

Advances in breeding efforts to increase the rate of genetic gains and enhance crop resilience to climate change have been limited by the procedure and costs of phenotyping methods. The recent rapid development of sensors, image-processing technology, and data-analysis has provided opportunities for multiple scales phenotyping methods and systems, including satellite imagery. Among these platforms, satellite imagery may represent one of the ultimate approaches to remotely monitor trials and nurseries planted in multiple locations while standardizing protocols and reducing costs. However, the deployment of satellite-based phenotyping in breeding trials has largely been limited by low spatial resolution of satellite images. The advent of a new generation of high-resolution satellites may finally overcome these limitations. The SkySat constellation started offering multispectral images at a 0.5 m resolution since 2020. In this communication we present a case study on the use of time series SkySat images to estimate NDVI from wheat and maize breeding plots encompassing different sizes and spacing. We evaluated the reliability of the calculated NDVI and tested its capacity to detect seasonal changes and genotypic differences. We discuss the advantages, limitations, and perspectives of this approach for high-throughput phenotyping in breeding programs.

## 1 Introduction

Climate change causes widespread changes in weather patterns and therefore poses new challenges for plant breeders (Stamp and Visser, 2012; Xiong et al., 2022). To strategically plan for future crop genetics, plant breeders must consider how to assess germplasm performance in locations that better represent their future environments – i.e. climate analogue sites – which are likely further from their research stations and possibly in another country or continent, where frequent data collection may be challenged by the availability of trained personnel, travel, logistics and equipment. In addition, multi-environment trials (METs) are needed to enable prediction of genotype reaction-norms (van Eeuwijk et al., 2019; Cooper and Messina, 2021). These prediction models tend to be based on markers, big data and machine learning approaches, and they strongly rely on a standardized, quality-controlled set of data from many different environments. Since the contribution of a gene to a trait can vary depending on environmental conditions, the results of genomic selection, genome wide association studies (GWAS) and other genomics-driven breeding and research methods will be more precise and relevant if run using phenomic data from numerous locations representing the diversity among growing environments (Korte and Ashley, 2013; Jarquín et al., 2014).

Accurately linking genotypes to phenotypes requires large populations of replicated genotypes, which can be costly to evaluate, especially at multiple locations (Furbank and Tester, 2011). Furthermore, bias due to differences in specifications of instruments or their handling, human error, as well as poor plot quality due to irregular emergence and soil heterogeneity can render big data analyses useless. These challenges limit the scalability of current phenotyping techniques across diverse environments, especially when linking the phenomic data to genomic data. Genetics-based breeding technologies, such as genomic selection, speed breeding and gene editing (CRISPR/CAS), offer ways to accelerate breeding, but their value is limited by the quality and relevance of phenotypic data. Consequently, standardized phenotyping of experiments or nurseries grown at different locations has remained a bottleneck for the use of phenomic data in genomic analyses (Crossa et al., 2021).

High resolution satellites may contribute to address this bottleneck, and have been recently tested for monitoring small plots (Tattaris et al., 2016; Sankaran et al., 2020; Sankaran et al., 2021). However, apart from being relatively costly, the resolution of the multispectral bands used to be coarser than 1 m. This changed with the launch of the Pleiades (Airbus, 2022) and SkySat (Planet, 2022a) constellations. The fleet of 21 high resolution (0.5 m) SkySat satellites became fully operational in the fall of 2020. Daily acquisitions attempts are now guaranteed, resulting in at least one cloud free image every 7 to 10 days for most regions on Earth. This opens up the opportunity to monitor and phenotype breeding plots across diverse environments over an entire growing season with identical measurement protocols.

## 2 Perspective: harnessing multi-temporal high resolution satellite images for monitoring breeding plots in diverse environments

Many of the variables collected in crop phenotyping can potentially be generated from satellite images. The SkySat sensors have 4 spectral bands: blue, green, red and infrared. They can be used to calculate the normalized difference vegetation index (NDVI), which is a measure of the amount of vegetation and its greenness, and other bio-physical parameters, including plot establishment, and various canopy traits, such as ground cover (fCover), leaf area and chlorosis (Jin et al., 2021). Using a time series of standardized images, date of emergence, end of leaf growth (which is an approximation of heading or tasseling date), and senescence or maturity can also be estimated (Jönsson and Eklundh, 2004; Pérez-Valencia et al., 2022). From a series of images covering the entire growing period, the performance of selected lines can be evaluated under specific weather conditions around the time that they occur, such as cold or dry spells and heat waves. In this context, satellite-generated phenotypic data from METs can be easily complemented with information on the dynamics of the environments retrieved from weather station networks or the global ECMWF Reanalysis products AgERA5 (Boogaard et al., 2020) and ERA5 (Hersbach et al., 2018). These products provide daily or hourly weather data at a resolution of either 10 or 30 km in close to real time, allowing better enviromics for the optimization of prediction models within the framework of the modern plant breeding triangle (Crossa et al., 2021; Resende et al., 2021).

Satellite images would enable breeders and researchers to monitor their field-plots in a single time-point (for each image), across a time-span (multiple images), and collect performance data on germplasm throughout a season at locations around the globe. In addition, since each satellite image covers an entire research field, genotypes across a field trial can be effectively compared without the potentially confounding effect of time (compared to physically carrying a hand-held tool to each plot in the field while ambient conditions are drifting). Plot level data collected by satellites can also be used to compare plot quality and to perform statistical correction for spatial heterogeneity in the field that can otherwise confound the expression of yield and other traits. They also allow for quality control and verification of reported data, such as date of sowing and management of the plots according to protocol. The use of satellite data will ultimately allow for the inclusion of larger nurseries (more lines) and more locations. Biases due to differences in instruments, human or other experimental errors will be reduced, resulting in standardized, multi-temporal data sets that allow for comparisons among sites in close to real time.

However, nursery plots for wheat and maize, as well as for other crops, tend to be relatively small. Plots tend to measure 2 or more meters in length, but plot width might be a bottleneck for the use of satellite images. For maize, breeders plant 1 or 2 rows, whereas for wheat, plots usually consist of 2 to 6 rows. This results in plots that tend to be between 0.7 m (one row of maize) to 1.2 to 1.6 m wide, which may pose some challenges for the use of 0.5 m satellite data to capture pure vegetation pixels and avoid mixed pixels affected by the signal from soil surfaces or neighboring plots. The native resolution, or ground sampling distance (GSD) of SkySat images depends on the view angle of the satellite among others. The resolution of the multispectral bands at nadir is 0.81 m for SkySat-3 to 15 and 0.72 m for SkySat-16 to 21 (Planet, 2022b). To align the satellite images with each other, they need to be orthorectified (Leprince et al., 2007). During the orthorectification process, the images are being resampled to a standard resolution of 0.5 m. Thus, a SkySat, or any other pixel of a satellite image, is not an exact representation of the area it covers on the ground (Saunier et al., 2022). Other technical factors such as radiometric calibration, atmospheric correction and the point spread function of the sensor can also affect the quality of the data, being especially relevant when using time series and multi-environment comparisons. To assess the potential and limitations of the use of SkySat images for phenotyping, we conducted separate field campaigns in Mexico and Zimbabwe.

## 3 Case study

We used time series of SkySat imagery to estimate the NDVI (NDVI_SAT_) from wheat breeding plots. The NDVI_SAT_ values were evaluated in terms of their reliability—i.e. capacity to detect genotypic differences, and how observed seasonal changes were related to crop phenology—and how they are affected by the plot size and spacing; all this while comparing NDVI_SAT_ to NDVI calculated from a UAV (NDVI_UAV_) at different moments during the growing cycle in wheat and maize, respectively.

**Figure 1:**
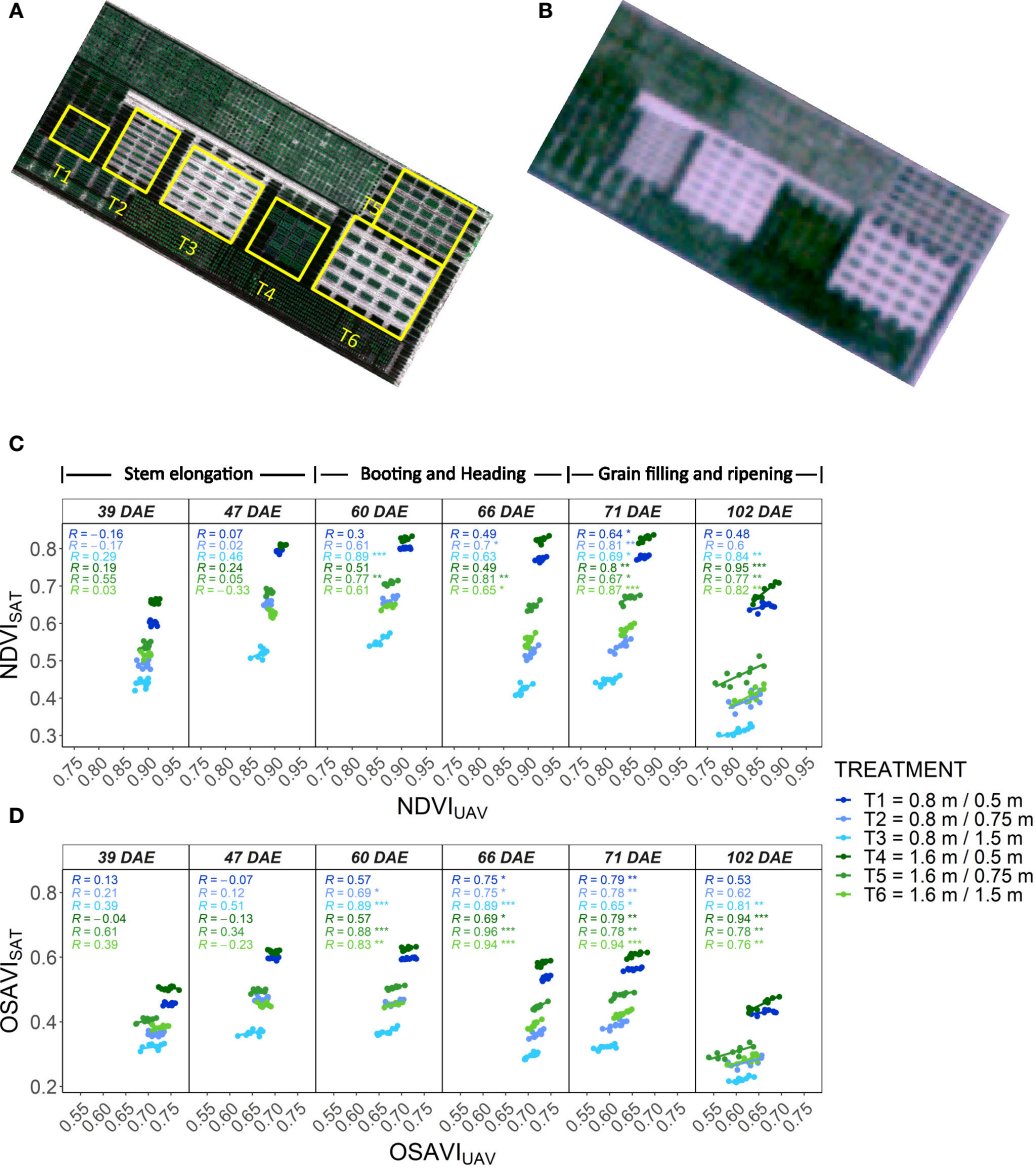
Assessment of SkySat images for the estimation of NDVI in wheat breeding plots with different size and spacing. **(A)** High-resolution RGB orthomosaic of the plot size and spacing experiment in wheat obtained from a UAV and boundaries of the different treatments. **(B)** RGB composite obtained from a SkySat image with a GSD of 0.5 m. **(C)** Correlations between NDVI_SAT_ and NDVI_UAV_ measured at different dates over the different treatments. **(D)** Correlations between OSAVI_SAT_ and OSAVI_UAV_ measured at different dates over the different treatments. NDVI and OSAVI values represent the best linear unbiased estimator (BLUE) computed individually at each measuring date for a given treatment based on a α-lattice design. Treatment description in the legend: “plot width/plot spacing”. The corresponding growth stage for each measuring date is indicated on top of Figure 1C.

A dedicated wheat experiment for assessing the effect of plot size and spacing in NDVI_SAT_ was planted at CIMMYT headquarters, Texcoco, Mexico (19.5338° N, 98.8428° W, 2,278 masl), under optimal growing conditions. A spring wheat panel comprising 10 genotypes from the Roots Anatomy panel was planted in six treatments resulting from the combination of two plot widths of 0.8 and 1.6 m (referred as small and big plots) and three spacings between plots of 0.5, 0.75 and 1.5 m in all directions (**Figures 1A, B**). The small and big plots had two and six rows of plants, respectively, and the same plot length of 2.5 m. Each treatment had an α-lattice design with two replicates, resulting in twenty plots per treatment. This experimental design is commonly used in breeding trials. The experiment was sown on 27 May and harvested on 5 October 2021. Aerial high-resolution multispectral images (GSD ~ 0.017 m) were collected at 25 m altitude using a RedEdge (Micasense, USA) multispectral camera mounted on a UAV (Matrice 100, DJI, China). The images were georeferenced using ground control points distributed along the field, and the spectral reflectance was calculated using a calibration target (Micasense, USA). A commercial software (Pix4D, Switzerland) was used to mosaic the images from which NDVI_UAV_ was extracted for each plot. The UAV images were collected across the cycle as close as possible to the satellite acquisitions (**Supplementary Table S1**).

Time series of SkySat multispectral images were collected over the wheat experiments starting from canopy closure. The acquisitions targeted a revisit frequency of 7 to 10 days. In order to limit BRDF effects (Royer et al., 1985) and distortion in GSD, maximum view angle was restricted to 16°. NDVI_SAT_ was calculated using the RED and NIR bands from the SkySat surface reflectance product (Planet, 2022b). Additionally, the Optimized Soil Adjusted Vegetation Index (OSAVI, Rondeaux et al., 1996) was calculated to mitigate the potential impact that soil brightness can have on NDVI, especially with larger plot spacing. A total of six satellite acquisitions were obtained during the cycle (**Supplementary Table S1**). For the extraction of NDVI and OSAVI, we first manually drew the plot boundaries based on an accurately geo-referenced UAV image. Using ArcGIS georeferencing tool, we then shifted the satellite images so that they would align with the plot boundaries. For this we employed sharp edges in the scenery as references, such as road corners and trial boundaries. After applying a 15 cm in-side buffer to the plot boundaries, we extracted the NDVI values with the R-library raster v3.6.3 using the normalizeWeights option, which accounts for the proportion of each pixel that falls in a polygon. The best linear unbiased estimators (BLUEs; Bernal-Vasquez et al., 2016) of the remote sensing data were calculated for each genotype using the R-package “asreml-R” version 4.1.0.160.

We also collected satellite images over maize breeding nurseries located in Muzarabani, Zimbabwe (16.3972° S, 31.0160° E, 498 masl). Three images were collected over the cycle starting from mid vegetative stage. However, UAV measurements were not available on site and satellite data could only be compared to NDVI readings measured with a hand-held optical sensor with adjustable arm (GreenSeeker, Trimble, USA). Therefore, details on the maize experiment and results are presented as supplementary material (**Supplementary Figure S1**).

A visual assessment of satellite images indicates that individualization of plots represents one of the challenges for extracting quality phenotypic data. Wheat plot boundaries were visually evident only for plots with a spacing of 0.75 m and 1.5 m (**Figure 1B**). In general, the increase of plot size and plot spacing resulted in higher and more significant correlations between satellite and UAV data, possibly due to better plot individualization (**Figures 1C, D**). In maize, plots were sown without spacing in between, hindering the visualization of plot boundaries. But the ranges as well as the edges of the experiment were clearly visible (**Supplementary Figure S1B**).

Given the satellite resolution and plot size, NDVI_SAT_ is expected to be affected by mixed pixels. The values of NDVI_SAT_ were much lower and showed a larger range between treatments in comparison to NDVI_UAV_ in all the dates (**Figure 1C**). While NDVI_UAV_ showed values close to saturation after canopy closure, NDVI_SAT_ ranged between 0.45 and 0.65, suggesting a degradation of the signal due to contamination from the neighboring bare soil. The OSAVI_SAT_ also showed lower values than OSAVI_UAV,_ except for the treatments with 0.5 inter-plot spacing where values were within the same range (**Figure 1D**). When plot spacing was increased, OSAVI_SAT_ decreased considerably to values much lower than those calculated from the UAV. A small inter-plot spacing facilitates the pollution of pixels by neighboring plots in the satellite data, which could explain the higher NDVI_SAT_ and OSAVI_SAT_ in plots with 0.5 m distance compared to wider plot spacing. In contrast, adding space between plots may imply a larger mixing of vegetation and bare soil spectra, decreasing the NDVI_SAT_ and OSAVI_SAT_. Conversely, the higher resolution of the UAV imagery can help avoid the effect of mixed pixels. However, increasing the plot spacing also decreased the values of both NDVI_UAV_ and OSAVI_UAV_ (although to a lesser extent than for NDVI_SAT_ and OSAVI_SAT_, respectively; **Figures 1C, D**). This suggests that mixed pixels may not be the only factor affecting the spectral signature when increasing the distance between plots. One possibility is that the larger spacing changes the illumination conditions within the plot due to more lateral light penetration.

Mixed pixels may also limit the capability of NDVI and other spectral indices to detect phenotypic variability from satellite imagery. In the small plots, the reduction of plot spacing resulted in lower average heritability values for NDVI_SAT_ and OSAVI_SAT_ (**Table 1**). In larger plots, the heritability values were higher than in small plots but there was not an evident effect of the plot spacing. This suggests that genotypic variability detected in larger plots may be affected by other factors such as heterogeneity within the plots, and that differences in heritability between treatments may be more related to weather and field conditions during data collection. In the UAV data, the heritability values were much higher than those from satellite data. However, the plot size and distance did not show a clear effect across dates. Instead, differences in UAV-based heritability between treatments and dates may be better explained by changes in environmental and operating conditions. Slight variations in factors such as illumination conditions, wind or view angle, among others, can affect the accuracy of the spectral measurements causing great impact in the calculated heritability.

**Table 1:** Changes in broad sense heritability related to plot size, spacing, and measuring date in days after emergence (DAE) for NDVI and OSAVI calculated from satellite (SAT) and UAV imagery.

			DAE 39	DAE 47	DAE 60	DAE 66	DAE 71	DAE 102	Average
NDVI	NDVI_SAT_	0.8 m / 0.5 m	0.00	0.04	0.00	0.00	0.00	0.00	0.01
		0.8 m / 0.75 m	0.23	0.00	0.00	0.52	0.44	0.56	0.29
		0.8 m / 1.5 m	0.33	0.33	0.48	0.86	0.63	0.50	0.52
		1.6 m / 0.5 m	0.00	0.00	0.25	0.75	0.75	0.90	0.44
		1.6 m / 0.75 m	0.25	0.31	0.38	0.00	0.15	0.82	0.32
		1.6 m / 1.5 m	0.52	0.00	0.00	0.63	0.41	0.81	0.40
	NDVI_UAV_	0.8 m / 0.5 m	0.87	0.81	0.95	0.97	0.82	0.89	0.88
		0.8 m / 0.75 m	0.79	0.73	0.83	0.84	0.81	0.82	0.81
		0.8 m / 1.5 m	0.93	0.83	0.95	0.91	0.87	0.82	0.88
		1.6 m / 0.5 m	0.91	0.85	0.96	0.98	0.97	0.96	0.94
		1.6 m / 0.75 m	0.58	0.15	0.72	0.86	0.47	0.76	0.59
		1.6 m / 1.5 m	0.65	0.50	0.92	0.87	0.84	0.93	0.79
OSAVI	OSAVI_SAT_	0.8 m / 0.5 m	0.12	0.41	0.00	0.43	0.00	0.00	0.16
		0.8 m / 0.75 m	0.28	0.00	0.00	0.59	0.51	0.62	0.33
		0.8 m / 1.5 m	0.31	0.24	0.52	0.00	0.64	0.47	0.36
		1.6 m / 0.5 m	0.00	0.00	0.29	0.77	0.84	0.86	0.46
		1.6 m / 0.75 m	0.37	0.36	0.53	0.00	0.14	0.86	0.37
		1.6 m / 1.5 m	0.57	0.00	0.00	0.72	0.60	0.89	0.46
	OSAVI_UAV_	0.8 m / 0.5 m	0.62	0.82	0.94	0.85	0.77	0.72	0.79
		0.8 m / 0.75 m	0.73	0.84	0.81	0.77	0.92	0.87	0.83
		0.8 m / 1.5 m	0.91	0.87	0.96	0.89	0.75	0.61	0.83
		1.6 m / 0.5 m	0.84	0.89	0.97	0.90	0.92	0.93	0.91
		1.6 m / 0.75 m	0.63	0.42	0.71	0.62	0.58	0.67	0.60
		1.6 m / 1.5 m	0.69	0.84	0.88	0.82	0.85	0.87	0.82

The time series of satellite images collected over the wheat experiment also depicted the influence of phenological stage on NDVI_SAT_ and its variability within each treatment. The phenotypic variability of NDVI and OSAVI from both platforms, and the correlations between them, were lower or not significant during the first two measuring dates (**Figure 1C**), coinciding with a time of highest biomass development during stem elongation. The correlations and variability increased later, from booting and during the grain filling, when decreases in green biomass and the onset of senescence may have maximized the differences in the spectral signature between genotypes. These phenological changes were more contrasting when plot spacing was larger, with the bigger plots always showing the highest heritability and the best correlations between both platforms on all dates.

## 4 Discussion

The consolidation of satellite platforms as tools for high-throughput phenotyping in breeding trials relies on many factors, among which the spatial resolution plays an important role. As expected, for plots with widths close to the sensor GSD low accuracies were observed. However, the results indicate that high resolution satellites hold promise for phenotyping plots commonly used in wheat (1.2 m) and maize (1.5 m) breeding.

In addition to the spatial resolution, other sensor specifications can have great impact in the usability of this data for plot phenotyping, and should be considered carefully for interpretation. Saunier et al. (2022) performed a deep analysis on the performance of the SkySat constellation, revealing a high signal-to-noise ratio, high geometric accuracy, and confirming that the spectral and spatial resolutions were compliant with the specification of Planet.

Nevertheless, the same study detected some sources of uncertainties, such as variations in the quality of the data coming from different sensors and changes in the spectral signature due to resampling. This has implications for the interpretation and comparison of time series data or data collected from different locations, especially for small plots, as images may be collected from different satellites and from a different view angle (i.e. differences in native spatial resolution). In this sense, the implementation of plot-level models to characterize trait changes over time, such as the ones suggested by Roth et al. (2021) and Pérez-Valencia et al. (2022), can be used to smooth time series of data, helping to reduce noise coming for systematic or random errors while improving the genotypic variability at key phenological stages. The atmospheric correction of SkySat imagery also presents limitations that can affect the quality of the data (Planet, 2022b; Saunier et al., 2022).

Modifying the plot spacing helped us realize the extent to which neighboring surfaces affect the plot spectral signature. We demonstrated that increasing plot spacing helps with the identification of individual plots and improves apparent heritability. Similarly, working with larger plots improved the accuracies. However, these solutions are not suitable for breeding programs, which tend to comprise several hundred plots. Pleiades Neo (Airbus, 2022), as well as the upcoming Pelican fleet of satellites (Planet, 2022a) will offer multispectral data acquired at a resolution close to 0.3 m. Hence, limitations set by the resolution are likely to become less of an issue. A remaining challenge will be the accurate delineation of the plot boundaries. This can be achieved with high-resolution UAV imagery, although a UAV may not always be available, especially in under-resourced programs or in remote regions. An accurate geometric layout of the plots, possibly with the help of an RTK GPS, together with placing fixed ground control points that can be identified in the satellite images, will facilitate the image-to-image registration and lining up with the plot boundaries. Nursery trials are generally sown in flat areas; hence a perfect alignment can be achieved by a simple shifting of the images, a process that can be automated.

The SkySat images were able to capture spatial heterogeneity in the small areas covered by our trials. Similarly, the temporal changes in the spectra agreed with the phenology of the crops. This, together with the possibility of capturing images on demand, opens the possibility of using the satellite information to characterize the field level spatial variability in models for prediction of genetic value (Araus and Cairns, 2014; Smith et al., 2021), and to remotely monitor the development and management of the trials for quality control at a low cost.

The successful collection of six satellite images during the rainy season in Central Mexico, while monitoring in parallel a maize trial in Zimbabwe, amply demonstrate the flexibility of this tool. With the imminent improvement of the spatial resolution, a remaining challenge will be the development and fine-tuning of operational procedures that ensure high quality, standardized data that will enable us to harness the benefits of the modern breeding triangle.

## Data availability statement

The data layers derived from the satellite images, such as NDVI and OSAVI, as well as all other data supporting the conclusions of this article, will be made available by the authors without undue reservation.

## Author contributions

FP, MZ-A and US conceived the idea, analyzed the data and wrote the manuscript. FP and MZ-A designed the experiments and collected the data. US managed and coordinated the satellite data acquisition. MR provided critical feedback. All authors contributed to the article and approved the submitted version.
