# Supplementary material for: Satellite imagery for high-throughput phenotyping in breeding plots
Source: Front Plant Sci. 2023 May 16;14:1114670. doi: 10.3389/fpls.2023.1114670 (PMC10227446; doi:10.3389/fpls.2023.1114670)
Supplement: Supplementary file 1 [file Table_1.docx]

**Supplementary Table S1:** Description of platforms, sensors and dates for NDVI measurements in the two locations.

| Location | Satellite GSD (m) | Validation platform | | |  | SkySat acquisition date | |  | | Validation dates | |
| --- | --- | --- | --- | --- | --- | --- | --- | --- | --- | --- | --- |
|  |  | Platform | Sensor | GSD (m) |  | Date | DAE |  | Date | | DAE |
| Mexico | 0.5 | UAV | Micasense  RedEdge | ~ 0.017 |  | July 10 2021 | 39 |  | July 7 2021 | | 36 |
|  |  |  |  |  |  | July 18 2021 | 47 |  | July 19 2021 | | 48 |
|  |  |  |  |  |  | July 31 2021 | 60 |  | July 30 2021 | | 59 |
|  |  |  |  |  |  | August 6 2021 | 66 |  | August 6 2021 | | 66 |
|  |  |  |  |  |  | August 11 2021 | 71 |  | August 18 2021 | | 78 |
|  |  |  |  |  |  | September 11 2021 | 102 |  | September 3 2021 | | 94 |
| Zimbabwe | 0.5 | Hand-held | GreenSeeker | ~ 0.015 x 0.61 |  | June 26 2021 | 24 |  | June 26 2021 | | 24 |
|  |  |  |  |  |  | August 11 2021 | 70 |  | August 10 2021 | | 69 |
|  |  |  |  |  |  | August 25 2021 | 84 |  | August 26 2021 | | 85 |
