# Supplementary material for: Satellite imagery for high-throughput phenotyping in breeding plots
Source: Front Plant Sci. 2023 May 16;14:1114670. doi: 10.3389/fpls.2023.1114670 (PMC10227446; doi:10.3389/fpls.2023.1114670)
Supplement: Supplementary file 2 [file DataSheet_1.pdf]

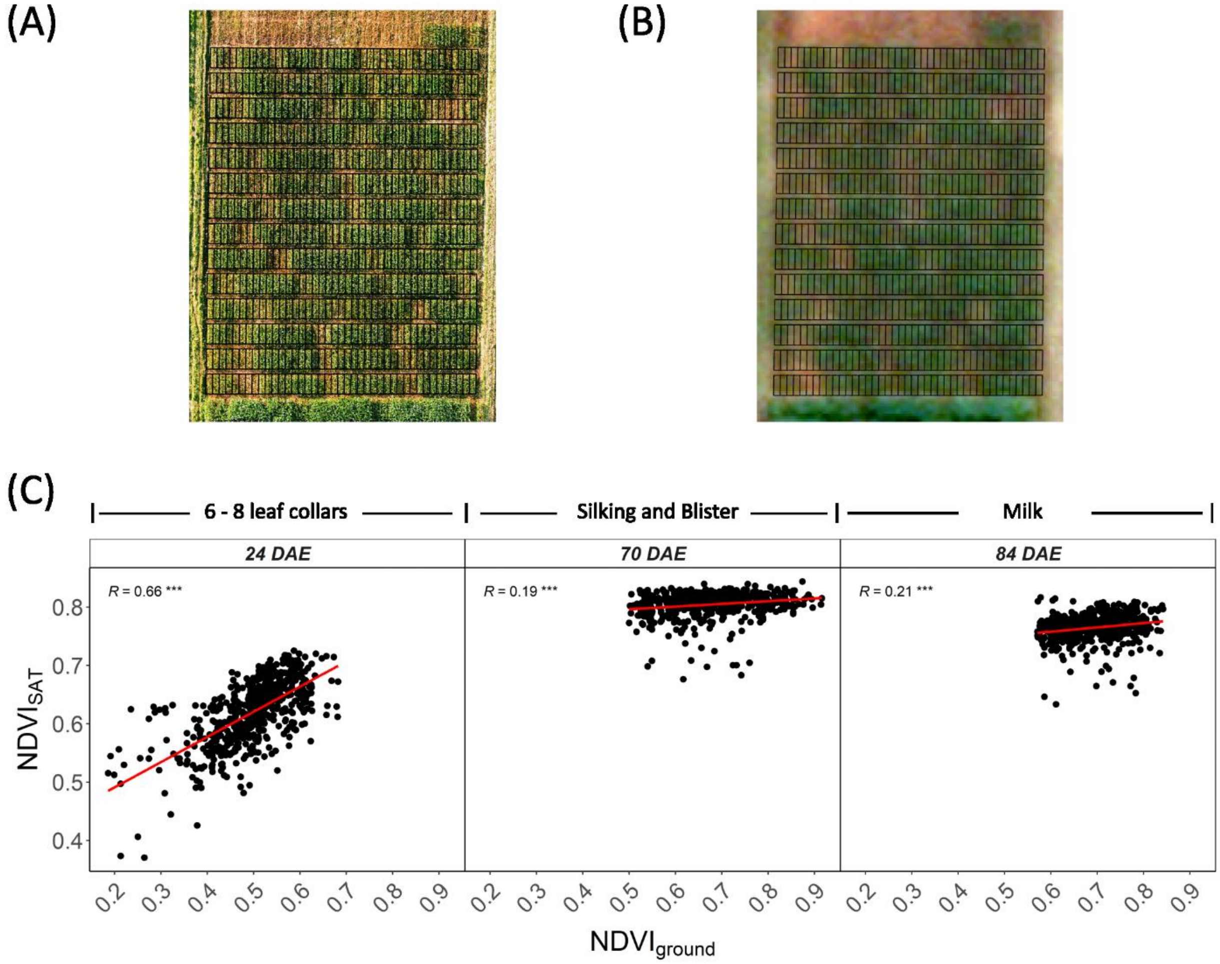

Supplementary Figure S1: Assessment of SkySat images for the estimation of NDVI in maize breeding plots. (A) High-resolution RGB orthomosaic of the maize nurseries obtained from a UAV. Black polygons represent the plots boundaries. (B) RGB composite obtained from a SkySat image. (C) Distribution of NDVI values calculated from satellite ($NDVI_{SAT}$) and GreenSeeker ($NDVI_{ground}$) images collected at different dates during the growing cycle. (D) Correlations between $NDVI_{SAT}$ and $NDVI_{ground}$ measured at different dates over the maize nurseries. The corresponding growth stage for each measuring date is indicated on top of Fig 1C.

Description of the maize experiment and results:

Three breeding nurseries encompassing a total of 533 plots were planted under optimal irrigation during the off-season at CIMMYT research station in Muzarabani, Zimbabwe (16.3972° S, 31.0160° E, 498 masl). The nurseries represented product profiles targeting different environments and they were part of two main breeding pipelines (early maturity and intermediate maturity). Each plot consisted of 2 plant rows of 4 m length with inter-row spacing of 0.75 m and in-row spacing of 0.25 m (Supplementary Fig S1 A and B). The nurseries were planted on 28 May 2021 and harvested on 25 October 2021. Weeds were actively controlled

during the vegetative stages. A hand-held optical sensor with adjustable arm (GreenSeeker, Trimble, USA) was used to measure $NDVI_{ground}$ at dates close to those of SkySat captures (Table S1). A RGB orthomosaic obtained by a UAV (DJI Phantom 4 Pro 2.0 equipped with a 20 MP DJI RGB camera) was used to identify plot boundaries in the satellite images (see description below).

The plots were sown without spacing in between, hindering the visualization of plot boundaries in the satellite images. But the ranges as well as the edges of the experiment were clearly visible (Supplementary Fig S1 B) and could be used for co-registration with the UAV image.

The discrimination between genotypes was good during the mid-vegetative stage 24 days after emergence (DAE) for both $NDVI_{SAT}$ and $NDVI_{ground}$. At that stage, there was a significant correlation between the two parameters (r=0.66, Supplementary Fig 1C). However, for the later stages, i.e. 70 DAE and 84 DAE, corresponding to one and three weeks after tasseling, respectively, the variation in NDVI was highly reduced, especially for $NDVI_{SAT}$, and a poor correlation between the two parameters was recorded. This reduction in the genotypic discrimination power of the SkySat images at later stages can be attributed to saturation resulting from maize canopy closure. As shown by Fitzgerald (2010), the NDVI measured with a GreenSeeker is less prone to saturation than when measured with a passive sensor. Other indices might be more suitable for satellite-based phenotyping of maize.

References:

Fitzgerald, G. J. (2010). Characterizing vegetation indices derived from active and passive sensors. *Int. J. Remote Sens.* 31(16), 4335-4348. doi: 10.1080/01431160903258217.
